# Treatment of heartworm (*Dirofilaria immitis*) disease in dogs in the USA: general findings and cost of disease

**DOI:** 10.1186/s13071-026-07487-x

**Published:** 2026-08-20

**Authors:** Kennedy Mwacalimba, Briana Ferguson, Christopher Brennan, Barbara Poulsen Nautrup, Jennifer Little, Kate Stiefelmeyer, Marina Moldavchuk, Ashley Mosko

**Affiliations:** 1 Outcomes Research, Zoetis, Parsippany, NJ USA; 2 Market Research, Zoetis, Parsippany, NJ USA; 3 EAH-Consulting, Aachen, Germany; 4 Kynetec, St. Louis, MO USA

**Keywords:** Heartworm disease, Heartworm prevention, Heartworm disease costs, Retrospective analysis, Transaction data

## Abstract

**Background:**

Heartworm (HW) disease is caused by the adult worms of *Dirofilaria immitis.* Treatment of HW disease is costly and often challenging. Therefore, the mainstay of HW disease management is to prevent the development of adult worms. The objective of this retrospective study was to use a transaction database to collect information and cost estimates on the prevention and treatment of canine HW disease in the USA.

**Methods:**

Anonymized transaction data from clinics throughout the USA were used. For the HW disease treatment group (TG) dogs were identified that had a transaction record for adulticidal therapy using melarsomine between August 2022 and July 2023. These dogs were tracked up to 95 days before the first melarsomine treatment for transactions given current American Heartworm Society (AHS) guidelines for the treatment of HW disease. Clinics with records of at least one dog with HW treatment were included. End of the tracking period was 5 days after the last melarsomine injection for the cost evaluation and 12-months for the evaluation of mortality. Included clinics were searched for dogs in the preventive group (PG), i.e., dogs that had a HW test between August 2022 and July 2023, followed by transaction of at least 8.9 HW preventive medication doses over the total observation period of 12 months, starting with the first transaction of HW preventive medication.

**Results:**

A total of 2425 dogs were included in the TG and 235,188 dogs qualified for the PG. The adherence to AHS guidelines was low in our study, with stipulated medications and treatments missing in many dogs. Total costs for an average dog with HW disease were calculated at $1457, not considering indirect or intangible costs. Costs of HW preventive medication in year-round purchase compliant dogs were estimated at $182.90. Dogs up to 9 years of age that were treated for HW disease had a significantly higher risk of mortality than dogs in the PG (*p* < 0.05).

**Conclusions:**

Costs of HW disease treatment are substantial, not considering the intangible effects on the animal and dog owner. The high costs and potential burden of disease underscore the benefit of year-round heartworm disease prevention.

**Graphical Abstract:**

**Figure:**
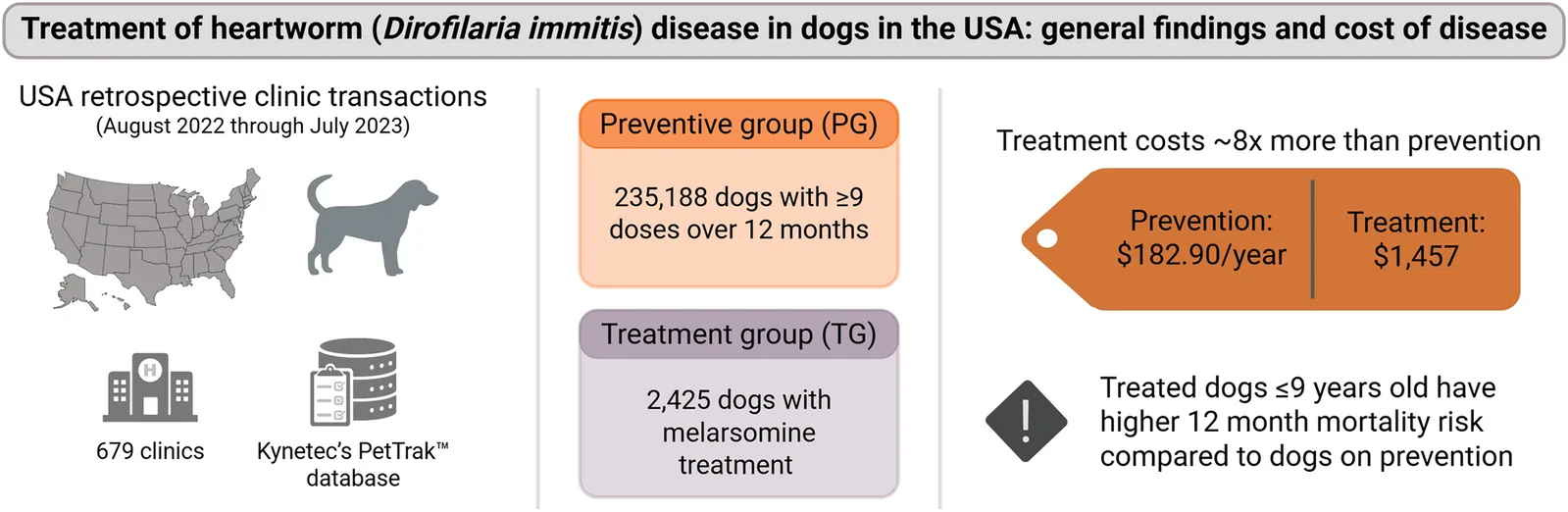

## Background

Canine heartworm (HW) disease is caused by the parasitic nematode *Dirofilaria immitis*, which is found in all states of the USA. Transmission occurs when mosquito vectors are feeding on a dog, thereby depositing infective *D. immitis* L3 larvae on the skin of the dog. After penetration of the animal’s skin, the larvae mature and migrate within the tissue, eventually reaching the pulmonary arteries and the heart, causing HW disease [1]. The American Heartworm Society (AHS) has developed guidelines for treating HW disease in dogs, with a last update published in 2024 [1]. The AHS recommends definitive adulticidal treatment with the injectable melarsomine dihydrochloride, an organic arsenical chemotherapeutic agent approved by the Food and Drug Administration (FDA) for adulticide treatment [2] (referred to as melarsomine). Following diagnosis, AHS recommends administration of a heartworm preventive with pretreatment with antihistamine and glucocorticosteroids if microfilaria are present, exercise restriction, and 28 days of doxycycline to eliminate *Wolbachia,* an endosymbiont bacteria harbored within *D. immitis* [1]. *Wolbachia* has shown to be necessary for embryogenesis, development and survival of the filarial parasite [3]. Eliminating the endosymbiont reduces the pathology in the dog which is associated with dead HWs after melarsomine treatment and disrupts HW transmission [1]. The AHS recommends an appropriate injectable or oral macrocyclic lactone heartworm preventive starting on the day of diagnosis, and continuing throughout the year. Melarsomine therapy should then begin 60 days after the heartworm disease diagnosis and pretreatment, with a total of three injections given on days 60, 90, and 91. In addition, the dog should receive an Environmental Protection Agency (EPA)- or FDA-approved ectoparasiticide for the entire treatment period and year-round thereafter. The dog should be retested for microfilariae on day 120, and then given an antigen test on day 365, which is 9 months after the last melarsomine injection [1]. The medical therapy over the first 3 months is presented in Table 1.

**Table 1:** Medical treatment of heartworm disease (days 0–91), as recommended by the American Heartworm Society [1]

Day	Treatment
0	• Doxycycline for 28 consecutive days • Heartworm preventive • EPA- or FDA approved ectoparasiticide • In symptomatic dogs only: prednisone • If microfilariae are detected: antihistamine and glucocorticosteroids (if not already on prednisone)
30	• Heartworm preventive (unless on injectable heartworm preventive) • EPA- or FDA approved ectoparasiticide
60	• First melarsomine injection • Pain control • Prednisone • Heartworm preventive (unless on injectable heartworm preventive) • EPA- or FDA approved ectoparasiticide
90	• Second melarsomine injection • Pain control • Prednisone • Heartworm preventive (unless on injectable heartworm preventive) • EPA- or FDA approved ectoparasiticide
91	• Third melarsomine injection • Pain control

The table considers medical treatment only. However, before starting medical treatment, heartworm positive dogs need to be diagnosed and verified by positive AG and microfilaria test. During medicinal treatment a rigid activity restriction is mandatory

Treating HW disease in moderately or severely affected dogs or in patients with concurrent diseases is often challenging [1]. Therapy, and the accompanying staging diagnostics and supporting therapy, is costly and not all owners are able to access treatment owing to the high costs, and adherence to recommended protocols is often incomplete. High-volume, low cost or vaccine clinics rarely offer the service [4]. The cost, complexity, and painful nature of adult HW disease treatment place a substantial burden on both dog owners and the dogs themselves, making prevention of larval maturation the mainstay of disease management. This preventive approach relies on a single drug class, the macrocyclic lactones (MLs) [5]. In the USA, monthly oral and topical products are available, either containing the macrocyclic lactone alone (ML-solo products) or in combination with other endo- or ectoparasitic agents (ML-combination products). Additionally, the injectable HW preventives ProHeart^®^ 6 and ProHeart^®^12 are available, providing protection over 6 or 12 months [6].

The objective of this study was to use a transaction database to collect general information and cost estimates on the prevention and treatment of HW disease in the USA.

## Methods

This retrospective study utilized transactional data recorded between August 2021 and July 2024 in Kynetec’s PetTrak™ database, which contained data from 679 veterinary clinics across the USA. The clinics were localized by the US Census regions.^1^ The transactional data included all sales captured in the clinics’ Practice Information Management System (PIMS) and were linked to a specific patient record, while maintaining patient anonymity. The data were used to identify and analyze treatment records of dogs that had received treatment for HW disease (treatment group, TG), as well as dogs that received nearly year-round preventive HW medication (preventive group, PG).

### Definition and tracking of treatment group patients

Dogs in the TG were identified by the transaction of a HW treatment medication, i.e., having a transaction record of melarsomine or the corresponding brand names in the USA (Diroban™, Zoetis, Parsippany, NJ, USA; or Immiticide^®^, Boehringer Ingelheim, USA) between August 2022 and July 2023. Dogs treated with alternative “slow-kill” protocols were excluded, as the AHS guidelines in effect at the time of treatment (published in 2018) did not recommend these protocols as standard first-line treatment and described them only “as a salvage procedure.” Clinics with records of at least one dog with HW treatment were included. According to AHS guidelines HW treatment should start with doxycycline and a ML preventive, and pretreatment before the first injection of melarsomine at day 60 after diagnosis [1]. Accordingly, dogs had to be tracked backward in the database. Transaction of all included dogs were tracked up to 95 days before the first melarsomine treatment for relevant transactions, including HW test, doxycycline, steroids, and/or antihistamine. If none of these transactions were found, the patient’s first HW treatment date minus 45 days was considered. End of the tracking periods was 5 days after the last melarsomine treatment for the evaluation of the cost of HW treatment. An additional analysis assessed the mortality of enrolled dogs during a 12-month observation period, while adulticidal therapy was ongoing.

### Definition and tracking of preventive group patients

The transaction records from included clinics, i.e., clinics with at least one transaction for HW adulticide, were evaluated for patients meeting the inclusion criteria for the PG. The clinics were checked for dogs that had a HW test between August 2022 and July 2023, followed by a transaction of a HW preventive product including ML-solo products or ML-combination products. The total observation period was 12 months in each dog, starting with the first transaction of HW preventive medication. A minimum of 8.9 doses of heartworm preventive medication over a 12-month period was used as an inclusion criterion. This threshold was empirically derived by Kynetec from aggregated PetTrak™ transaction data to represent dogs considered near-compliant with year-round heartworm prevention.

### Retrospective analyses

The database was used to collect general findings of HW prevention and treatment in the USA. Transaction data of included dogs in the TG and PG were used to compile specific findings, total costs, and costs of HW treatment and nearly year-round preventive medication. Finally, the mortality of dogs which had undergone HW treatment was compared with the death rate in dogs in the PG.

### General findings of HW treatment and prevention

The database was also used to generate general descriptive findings on HW treatment, including the number and percentage of clinics with dogs treated for HW disease and the average HW disease patients per clinic. Additionally, the number and percentages of dogs receiving any HW preventive medication was recorded. All results were split by Census regions and divisions.

### Specific findings of HW treatment and prevention

This analysis considered only dogs included in the TG and PG. For dogs in the TG, the following parameters were recorded: number of dogs and average age, records of HW test and transactions before melarsomine treatment, diagnostic procedures and hospitalizations, the number of melarsomine treatments, and average treatment duration from start of the tracking period until 5 days after the last HW adulticide treatment.

In the PG the number of dogs was recorded, split by HW preventive type (ML-solo or ML-combination product), as well as the average moving annual total (MAT; i.e., the cumulative total for the most recent 12-month period) dose.

### Costs of HW disease treatment and prevention

First, costs were considered during the tracking period, including all transactions, split into costs that could be directly related to HW disease treatment and additional costs. The costs of transactions specifically assigned to HW treatment (HW disease specific costs) included melarsomine therapy, as well as supportive treatments (doxycycline, antihistamines, antiemetics, steroids, and pain and sedation, the latter including NSAIDs, opioids, gabapentin, and sedatives), diagnostics (HW test, radiology, echocardiography, electrocardiography, and others), hospitalization, and other fees (examination, biohazard, and injection fees). Additional costs included all other transactions recorded over the tracking period.

An additional analysis aimed to evaluate services and medications that could be directly related to the melarsomine injection. Therefore, transactions of HW disease specific costs were considered that accrued on the day of injection or up to five days after the melarsomine administration.

The costs of preventive HW medication were collected over the 12-month observation period (MAT), split by ML-combination products and ML-solo preventives.

A final analysis compared the total cost of care between TG and PG, accrued over a 12-month period, looking at all transactions and visits. The data was segregated by US Census regions.

### Mortalities within a 12-month observation period

Mortalities included spontaneous deaths and euthanasia. Beginning with the start of tracking period, deaths were collected in both groups over a 1-year observation period. Mortality rates were split by age groups, considering the following age segments: < 1 year, 1–3 years, 4–6 years, 7–9 years, 10–12 years, and 13 + years. In cases where the age of the dog could not be determined, it was assigned to the age group “unknown.”

## Results

### General demographic data of HW treatment and prevention

The entire database, with entries between August 2021 and July 2024, was used to evaluate prevention and treatment patterns of HW disease in different regions of the USA.

Out of a total of 2.4 million active patients, 41% were tested for HW disease. This amounted to a total of 983,773 patients. The New England region had the highest percentage of screened patients at 64%, while the Mountain and Pacific regions had the lowest screening rates, both at 20%.

When looking at patients on heartworm prevention, the data showed that 37% received HW protection, totaling to 890,088 patients for whom at least one HW preventive transaction was recorded. East North-Central in the Midwest region had the highest proportion of patients on HW prevention at 44%, while the Pacific and Mountain regions again showed the lowest levels of protection with 19% each.

Of the 679 clinics included in the database, 540 (79.5%) had records of HW disease treatments, with significant variation across different regions. In New England all clinics (100%) included in the database had at least one dog treated for HW disease. Corresponding rates in the East, South-Central and South Atlantic regions were 94% and 90%, respectively. By contrast, only 47% of the clinics in the Mountain region, and 37% in the Pacific region had records of melarsomine treatment (Table 2).

**Table 2:** Number of clinics and patients with transactions of heartworm disease treatment or preventives per Census Region

Census region	Division	Treatment group			Preventive group
		Clinics with Dogs treated for HW *n* (%)	Dogs treated for HW *n* (%)	Average HW Patients per Clinic	Dogs receiving HW preventive *n* (%)
Northeast	New England	9 (100%)	26 (0.1%)	2.9	9825 (41%)
	Middle Atlantic	41 (77%)	160 (0.1%)	3.9	58,699 (32%)
Midwest	East North Central	125 (79%)	490 (0.0%)	3.9	221,548 (44%)
	West North Central	58 (82%)	322 (0.1%)	5.6	97,922 (38%)
South	South Atlantic	142 (90%)	1209 (0.2%)	8.5	217,964 (40%)
	East South Central	68 (94%)	778 (0.3%)	11.4	107,942 (40%)
	West South Central	61 (92%	846 (0.3%)	13.9	106,536 (43%)
West	Mountain	8 (47%)	30 (< 0.1%)	3.8	14,863 (19%)
	Pacific	28 (37%)	52 (< 0.1%)	1.9	54,789 (19%)
Total		540 (80%)	3913 (0.2%)		890,088 (37%)
Included clinics and patients					
Total		418	2425		235,188

The database analysis included all entries in the database between August 2021 and July 2024. Included clinics had to have at least one dog with a transaction record of melarsomine treatment between August 2022 and July 2023

HW, heartworm

Out of the total active patients, 3913 (0.2%) received melarsomine. The highest treatment rates were recorded in the East South-Central (0.3%) and West South-Central (0.3%) regions. Other regions, such as the Mountain and Pacific regions, saw much lower treatment rates (< 0.1%). These proportions do not account for HW positive patients that did not receive treatment with melarsomine.

The average number of HW disease treatment patients per clinic also varied considerably between regions. The West South-Central region saw the highest average, with 13.9 patients per clinic, while the East South-Central region followed with 11.4 patients per clinic. In the Pacific region, on average only 1.9 patients were treated, thus having the lowest number of HW patients per clinic (Table 2).

### Specific characteristics of the treatment and prevention groups

In total, 2425 dogs were identified in 418 clinics, receiving HW disease treatment between August 2022 and July 2023, thus fulfilling the inclusion criteria for the TG. There were 235,188 dogs that fulfilled the inclusion criteria for the PG, being identified in the 418 clinics included in the analysis. Average age was 3.9 years in the TG and 5.5 years in the PG.

From the 2425 patients in the TG, 2280 (94.0%) had an initial transaction associated with HW treatment before the melarsomine injection. The average time between first transaction and the first melarsomine injection was 50.3 days. However, 145 patients (6.0%) lacked any relevant transactions before melarsomine treatment. A total of 1786 dogs (74%) had a record of HW test and 1705 (70%) dogs had transactions for doxycycline. Steroids were prescribed to 1957 dogs (81%) and 234 dogs (10%) received antihistamines.

The number of melarsomine injections ranged from one to five, with most dogs receiving two (51.8%) or three (28.0%) injections. Overall, 19.5% of patients received only one injection and a small percentage of dogs received four (0.6%) or five (0.1%) melarsomine treatments (Table 3). Pain control was administered to 1676 dogs (69%). Radiology, electrocardiography, and echocardiography were used diagnostically in 41%, 1%, and < 1% of the dogs, respectively. Seventy percent of all included dogs were hospitalized. Average treatment duration was 81 days across all patients.

**Table 3:** Number and time of melarsomine injections, as well as deceased patients split by number of treatments received

No. of melarsomine treatments	Patients		Average days between start of tracking and last HW treatment	Deceased patients	
	No	% of total patients		No	% of total patients
1	472	19.5	35	36	7.6
2	1257	51.8	83	73	5.8
3	680	28.0	89	31	4.6
4	14	0.6	113	0	0.0
5	2	0.1	246	0	0.0
Total	2425	100	76	140	5.8

HW, heartworm

In the PG, 164,924 dogs received ML-solo products and 80,140 dogs received ML-combination products. However, some dogs received products of both groups, resulting in a higher number of entries than dogs included (235,188). In all patients, the average MAT dose was 11.8, thereby exceeding the minimum number of doses required for inclusion (Table 4).

**Table 4:** Costs of nearly year-round HW preventive medication, stratified by number of active ingredients (ML-solo versus ML-combination products) and formulation

Application	No. of patients	Average MAT doses	Average cost per dose ($)	Average MAT costs per application type ($)
ML-combination products				
Oral	77,971	11.0	27.70	304.70
Topical	2486	9.9	22.50	222.75
*Average*	*80,140*	*11.0*	*27.60*	*303.60*
ML-solo products				
Oral	71,150	11.2	10.20	114.24
Injection	96,274	12.0	10.40	124.80
*Average*	*164,924*	*11.7*	*10.30*	*120.51*
Average All ML products	235,188	11.8	15.50	182.90

Patients with different brand purchases in MAT could be counted in multiple groups. Thus, the total number of the patients abbreviates from the sum

MAT, moving annual total; ML, macrocyclic lactone

### Cost of HW disease treatment

The costs of HW disease treatment included all transactions during the tracking period, being split in costs that could be directly related to treatment of HW disease and additional costs accrued. Total costs in dogs treated for HW disease were calculated at $1457, with $1124 being defined as specifically related to HW disease treatment. The total costs split by cost groups are displayed in Table 5, exclusive of HW preventives. When preventives are included, i.e., the average costs of $183 as calculated in section “Cost of year-round HW prevention,” the total costs are calculated to $1640 and $1307, respectively.

**Table 5:** Breakdown of heartworm treatment costs

Service/medication type	Patients	% of total patients	Cost per patient	% of total costs
HW medication	2425	100%	$560	38.5%
Additional medication	2309	95%	$154	10.1%
Antiemetics	211	9%	$74	0.4%
Antihistamine	234	10%	$43	0.3%
Doxycycline	1705	70%	$83	4.0%
Pain treatments	1676	69%	$65	3.1%
Steroids	1957	81%	$40	2.2%
Exam / consultation	1923	79%	$110	6.0%
Diagnostics	2004	83%	$276	15.7%
Hospitalization	1697	70%	$141	6.8%
Additional fees	491	20%	$19	0.3%
HW treatment costs	2425	100%	$1124*	77.1%*
Additional costs accrued over the tracking period			$333	22.9%
Total costs (MAT)	2425	100%	$1457	100%

HW medication was melarsomine; Diagnostics included HW test, echocardiogram, electrocardiogram, radiology, and other; Additional fees included biohazard fee and medication/injection fee

^*^In nine patients negative transactions were recorded, including credits from manufacturers for product guarantees, thus reducing the sum of average costs

HW, heartworm; MAT, moving annual total

The primary cost driver was the melarsomine injection used to kill adult HW. This therapy alone represented 38.5% of the total cost, equating to an average of $560 per dog. The costs included not only the cost of the medication but also the administration of the drug during veterinary visits. However, in some cases, medication administration/injection fees were invoiced separately. Diagnostics accounted for 15.7% of the total cost, averaging $276 per patient. Dogs undergoing HW disease treatment often received additional medications, such as antihistamines, antiemetics, doxycycline, steroids, and pain management drugs. These medications represent about 10.1% of the total cost, or on average $154 per dog.

Approximately 70% of dogs treated for HW disease required hospitalization at some point during treatment. This cost, which includes monitoring and boarding, contributed to about 6.8% of the total costs, averaging $141 per patient.

Additionally, medications and services directly related to the melarsomine injection were identified. All dogs that were prescribed pain medication received it at the day of melarsomine injection or up to 5 days after. The same applied to the prescription of antiemetics, biohazard fees and injection fees. In most dogs receiving steroids and antihistamines, administration of these medications were also related to the timing of melarsomine injection. Of all dogs that received steroids or antihistamines, 96% and 72%, respectively, received these medications within 5 days of melarsomine administration.

### Cost of year-round HW prevention

The minimum criteria for a dog to qualify for the PG was an average of 8.9 MAT doses of ML-solo or ML-combination formulations. Of all included dogs, the mean MAT ML doses were calculated at 11.8. The highest MAT doses were achieved with the injectable ML preventive (12.0 doses), whereas the lowest MAT dose was recorded for topical ML-combination products (9.9).

Oral ML-combination products averaged $27.70 per dose whereas the topical ML-combination products were $22.50 per dose. Average cost per month was $10.40 for the ML injectable product and $10.20 for the oral ML-solo products. All results are presented in Table 4.

Considering an average MAT dose of 11.8 for all products for comparability, total annual preventive costs would be $326.86 and $265.50 for the oral and topical ML-combination products, respectively, whereas total annual costs for the ML-solo products would be $120.36 (oral) and $122.72 (injectable). Over both product groups, average annual costs would be $184.08, considering an average MAT dose of 11.8.

### Average cost of total care over a 12-months period

When examining the broader cost of veterinary care, i.e., costs associated with HW disease treatment or prevention and all other costs recorded over the MAT, dogs in the TG incurred on average $1948, with a range of $1872–2881. The lower cost bound was found in the South region and the highest average costs in the Northeast region. By contrast, dogs on prevention incurred on average $1175 annually, with lowest average costs ($1126) recorded for the Midwest region and highest costs ($1380) for the Northeast region.

### Mortalities observed over one year in the TG and PG

**Fig. 1:**
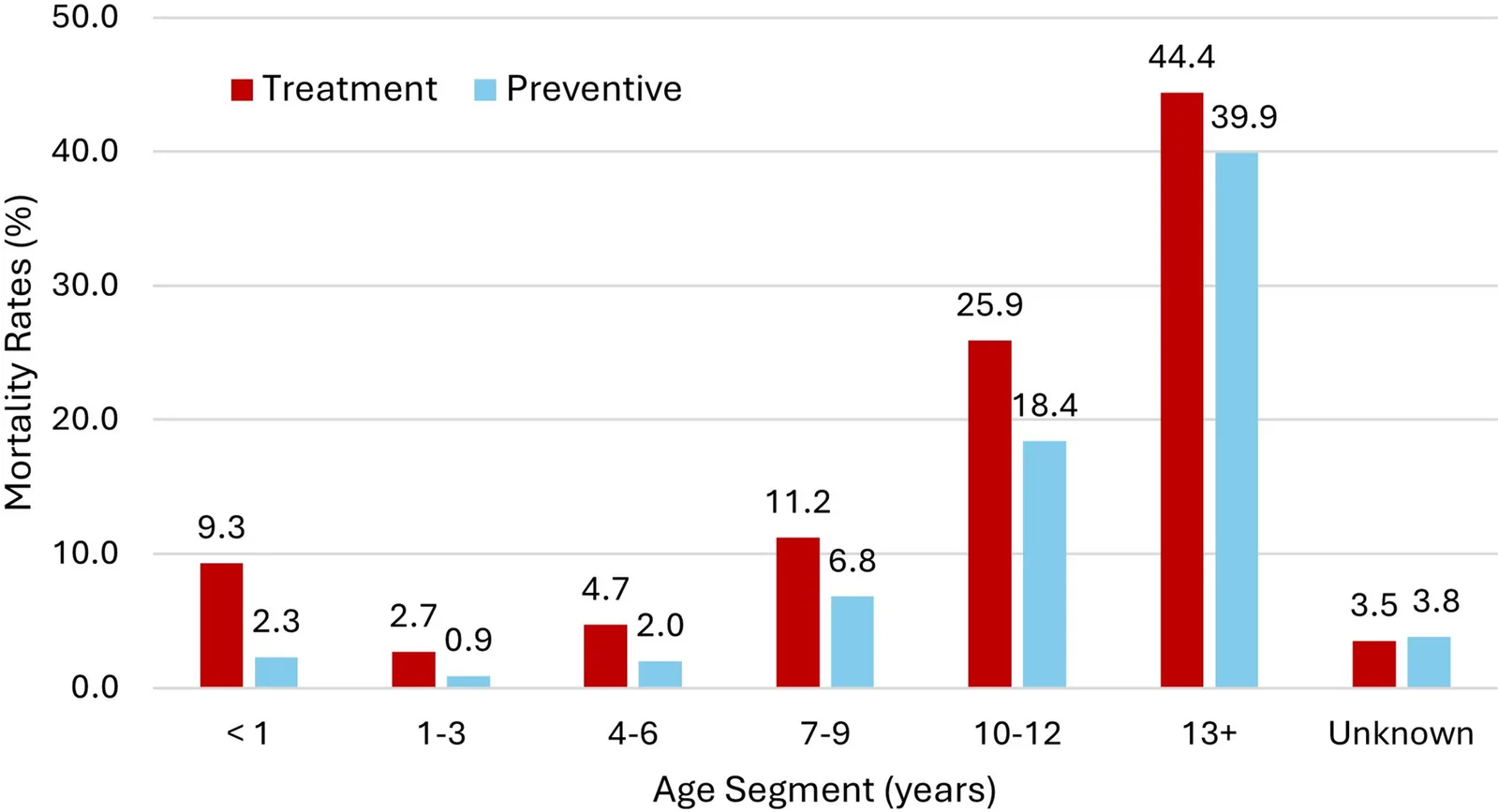
Mortality rates in the treatment and preventive group, split by age segment. In case the age was not known, the dogs were classified in the “unknown” group

**Fig. 2:**
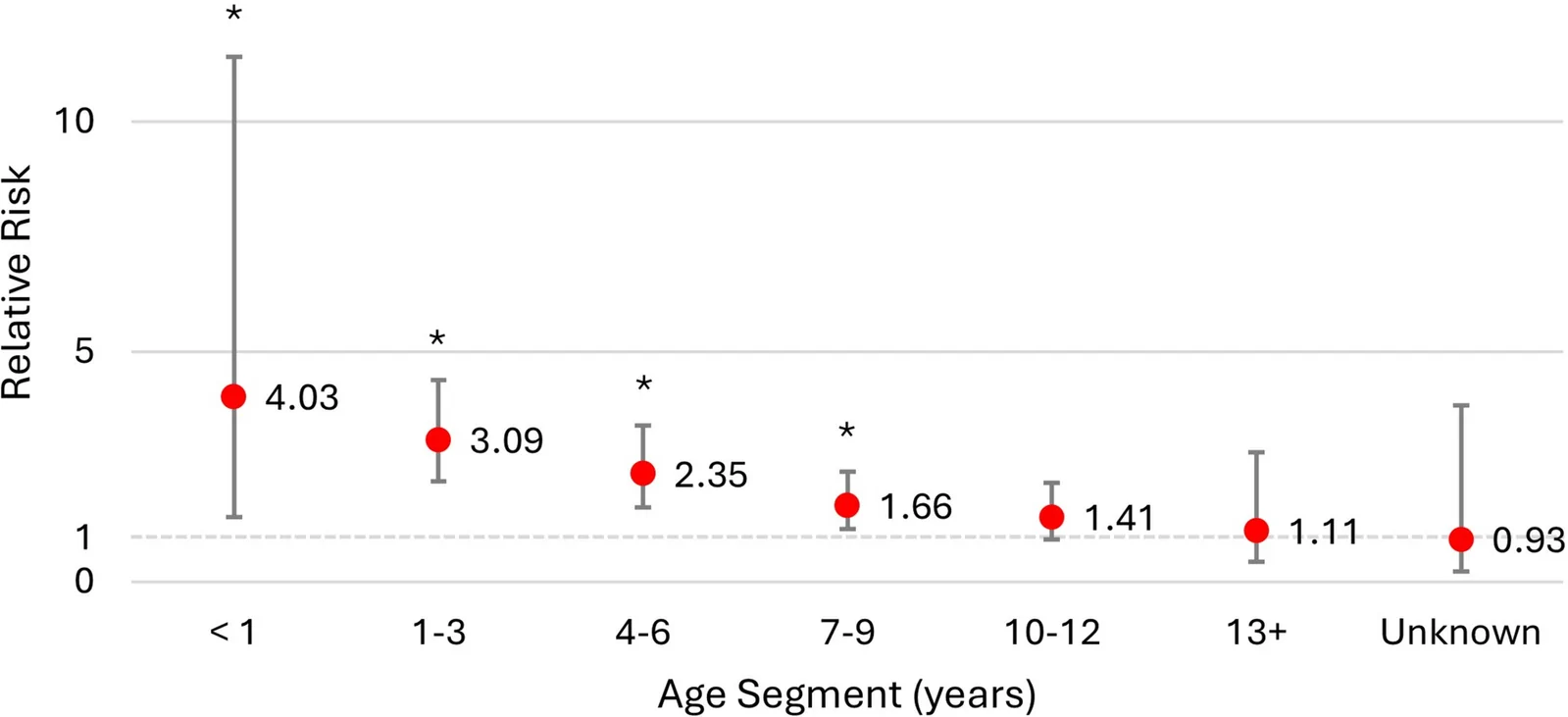
The relative risk of death between the treatment and prevention groups, split by age segment. *p < 0.05

Overall mortality rates were 5.8% and 6.1% in the TG and PG, respectively. As the age distribution was not similar between the two groups, mortality rates were analyzed by age groups. The highest mortality rates in both groups were seen in the age group 13 + years (44.4% in the TG and 39.9% in the PG). The highest difference between TG and PG were seen in the age segment < 1 year (9.3% versus 2.3%, respectively). Between the age segments 1–3 years and 13 + years, there was a steady increase of mortality rates in both groups (Fig. 1). The relative risk of mortality was statistically significantly (*p* < 0.05) higher in dogs in the TG versus PG between < 1 year and 9 years (Fig. 2).

Another analysis considered the mortality rates in relation to the number of treatments. In the group of dogs that that received one melarsomine treatment, the mortality rate was 7.6%, whereas in the groups of dogs that received two or three melarsomine treatments, mortality rates were 5.8% and 4.6%, respectively. No deaths were recorded in dogs receiving four or five treatments. (Table 3).

## Discussion

### General comment

The AHS guidelines cited in this study were published in 2024, i.e., after the treatments recorded in our database had been administered. However, the general recommendations relevant to our analysis have remained largely unchanged. One exception is the “slow-kill” protocol, which the current guidelines recognize as a treatment option in selected cases, including dogs with contraindications to melarsomine, dogs with a grave prognosis, and working dogs in which adequate activity restriction is not feasible. However, this protocol was not considered in our analysis, as it was not acknowledged as standard first-line treatment but only “as a salvage procedure” at the time of treatments.

### General findings of HW disease in dogs in the USA

The data collected from 679 veterinary clinics between August 2021 and July 2024 were used to compile general findings of HW disease across various regions of the USA. Overall, 41% of all active patients were tested within the 1-year observation period for HW disease, although the AHS recommends annual screening for all dogs over 7 months of age [1]. Of all clinics included in the database, 540 (80%) had records for treatment of HW disease. However, it is not known if the other clinics did not offer the treatment within their services, actually had no dog identified positive for HW disease, or had dogs tested positive that were not treated with melarsomine for economic or other reasons. Relevant variations in patient screening rates, HW disease treatment rates, and patients on preventive medication were recorded for the different Census regions. In general, the distributions of clinics with highest and lowest rates reflected the distribution of cases on the HW incidence map, published by the AHS for 2022^2^. According to the AHS, most HW cases were located in and adjacent to the lower Mississippi Delta, with Mississippi, Louisiana, Texas, Alabama, and Arkansas representing the states with the highest density of diagnosed HW cases [7], where the climate, wetlands, and mosquito proliferation favor the transmission of *D. immitis* [8]. However, the number of HW disease patients per clinic was much lower in our study than the number of cases per clinic on the HWD incidence map of the AHS for 2022, which displays areas with more than 100 reported cases per clinic. Incidences published by the AHS represented HW disease cases, reported from participating clinics. The higher number of cases per clinic in the AHS survey might result from selection bias, if clinics with high numbers of HW disease cases were overrepresented in the pool of reporting clinics. Additionally, the cases reported to the AHS included all positive dogs, thereby also including those that were diagnosed but not treated for different reasons, or dogs that were treated with the so called “slow-kill” protocol. 

The percentage of patients receiving melarsomine treatment was relatively low in our study, with 0.2% of the total active patients. The American Veterinary Association estimated the number of dogs in 2024 in the USA at approximately 90 million.^3^ Given the published figure of over 1.2 million canine HW disease cases in 2021^4^, this corresponds to 1.3% of the dog population testing positive for HW disease that year. The lower percentage in our study, however, might be owing to the same reasons as assumed for the lower numbers of cases per clinic.

The mean percentage of patients receiving any HW prevention was 37% over all regions. A comparably low rate (33.3%) of medicalized dogs in the USA receiving one or more doses of HW preventives annually has been reported previously [9].

### Specific findings of HW treatment and prevention

A HW test was recorded in only 74% of dogs before melarsomine treatment; a positive antigen test is mandatory for diagnosis, whereas diagnostics, such as radiography, echocardiography, and ultrasound evaluation, are useful for confirming and staging the severity of HW disease [1]. It is unclear whether dogs without a recorded heartworm test truly did not receive a test, whether a test was performed but not recorded, or whether only microfilaria testing was carried out.

Additionally, it cannot be ruled out that in some dogs the time between test and start of melarsomine treatment exceeded the tracking period used in our study. In a previous study, 11% of dogs did not receive their HW disease treatment within 10 months after a positive test, including dogs which were not compliant with doxycycline therapy and therefore were asked to return upon completion [4]. Although misdiagnosed cases of HW disease cannot be ruled out, our study did not intend to evaluate the efficacy of treatment, but to evaluate actual treatments conducted in veterinary clinics, thereby reflecting the actual situation in veterinary routine.

The start of treatment with doxycycline was only documented in 70% of dogs, despite being stipulated by the AHS. Doxycycline eliminates *Wolbachia*, an endosymbiont bacteria harbored within *D. immitis*, thereby reducing the pathology associated with dead HWs and disrupting HW transmission. Accordingly, melarsomine protocols without administration of doxycycline have resulted in higher complication and mortality rates [1].

It is known that the injection of melarsomine is painful, as it is a highly irritating drug known to potentially cause substantial tissue damage [4]. Nevertheless, pain medication was only recorded in 69% of dogs. We do not know whether the recommended pain treatment was given but not recorded, or whether it was omitted for various reasons, such as cost.

Only 28% of dogs received the recommended number of three melarsomine treatments. Nearly 52% of dogs received two melarsomine treatments and almost 20% only one melarsomine injection. There are numerous potential reasons why an owner might not return for a second injection, including forgetfulness, adverse effects observed after the first injection, financial constraints, deviation from AHS recommendations by the attending veterinarian, or the death of the patient. Although the outcome is not known, it can be rationally assumed that treatment was not 100% effective in these dogs as the first dose kills only approximately 50% of adult worms [10]. If these dogs were not cured, and still harbored both male and female worms, they would continue having microfilaria and be *D. immitis* reservoirs [4]. A small percentage of dogs received four (0.6% of total patients) or five (0.1% of total patients) melarsomine injections.

Altogether, many dogs in our study did not receive the HW disease treatment as recommended by the AHS. Owner’s financial concerns have been frequently reported as reason for modifications of the recommended HW disease treatment regimen [11].

Regarding the dogs in the PG, the minimum of HW preventives was set at 8.9 for inclusion, to generate cost data for purchase compliant dogs. However, the actual average MAT doses were 11.8, thus the dogs in the PG can be considered as purchase compliant. It should be noted, however, that the compliance in this group does not reflect the purchase compliance over all dogs in the USA, which has been estimated at 7.3 with monthly oral or topical ML preventives, considering dogs that received at least one prescription of ML preventive [6]. Accordingly, the purchase compliance in our study does not reflect the average preventive doses dogs receive in the USA. However, the inclusion of compliant dogs was meant to allow the calculation of costs for a year-round protected dog rather than calculate the average costs in dogs with lower compliance, which are at risk of HW disease.

### Cost of HW disease treatment

Costs were driven by multiple factors, reflecting the complex and resource-intensive nature of managing the disease.

Over the tracking period, average total costs in dogs treated for HW disease, also considering the recommended HW preventive medications were calculated at $1641, with $1308 including only the medications and services defined as directly related to treatment, i.e., being recommended by the AHS for HW disease treatment. As such, costs of HW treatment were much higher than those estimated between $225 and $614 in a low-cost high-volume outpatient care setting in Texas. However, in the two included clinics, the AHS protocol was modified to mitigate financial barriers, i.e., to deliver an effective and safe melarsomine therapy at a more affordable cost to pet owners [4].

Although considered in our study, antiemetics were not included in the AHS guidelines. Severe gastrointestinal symptoms, such as vomiting, inappetence and diarrhea, have been reported with the use of doxycycline [12] and melarsomine [4], and might explain the prescription of antiemetics in treated dogs. However, the percentage of antiemetics’ contribution to total costs was very low (0.4%) thus not relevantly influencing our results.

Total treatment costs as calculated in our study can be regarded as significant underestimation of those costs that would accrue in dogs receiving a fully compliant treatment regimen, as recommended medication was not prescribed to all dogs and only 28% of all dogs received three melarsomine injections. Although average costs also include dogs that received four or five treatments, the percentages were negligible in terms of contributing to average costs compared with dogs receiving only one or two treatments. As melarsomine treatment was the main cost driver, the costs of treatment according to AHS can be rationally assumed to be much higher. Additionally, in our study only direct costs of treatment were considered. Indirect costs, such as travel expenses to the veterinary clinic and days off work, that might apply if dog owners need to take days off to care for their pets at home or to spend in the veterinary clinic, have not been captured in our analysis of transaction data. The same is true for euthanasia costs in dogs euthanized owing to HW disease and additional medication, such as further HW tests after melarsomine treatment to confirm negativity. Another important type of costs also has not been considered, the intangible costs. Intangible costs—*per definitionem*—cannot be expressed easily in monetary units but can be assumed to be a significant burden with HW treatment. They include the emotional burden associated with managing HW disease. Dogs receiving HW disease treatment require careful monitoring, adherence to medications, and restrictions on physical activity to prevent complications. The emotional strain can be particularly high, as owners worry about potential complications and the long recovery process. Ensuring that the dogs are kept calm and inactive, might disrupt the pet owner’s normal routine, potentially leading to frustration or difficulty adjusting their daily schedules, further adding to the burden of care. Last but not least, not to forget the reduced quality of life of the dog during the long term, stressful and painful therapy.

### Cost of HW prevention

Average per-dose cost for ML-combination products was about twice that of ML-solo preventives (Table 4), consistent with the inclusion of additional active ingredients for endo- and ectoparasite control. The average MAT costs for all HW preventive products were calculated at $183.90, thus being considerably lower than treatment costs that accrue in case of HW disease.

In the PG, we only considered medication costs, but not consultation costs, which might underestimate the total preventive costs, as at least one HW test was mandatory for inclusion. However, it can be reasonably assumed that follow-up purchases were done without consultation or alongside consultation for other reasons.

Dogs should receive heartworm preventives year-round for life, rather than being limited to a single year. Therefore, the ongoing costs of prevention would be similar in both groups.

### Average cost of total care over a 12-months period

Over a 12-month period, dogs in the PG incurred about 60% of the total veterinary costs of dogs in the TG ($1175 versus $1948). After removing HW-specific spending in each group, the remaining “other-care” spend in the TG was roughly half that of the PG (~$491 versus ~$992). This difference is consistent with two non-exclusive explanations: (i) the PG is a more medicalized cohort by design (near-compliant HW prevention as a proxy for greater uptake of wellness/preventive services), and (ii) the economic and emotional burden of HW treatment may reduce use of other services in the TG. Because PG inclusion required preventive purchase compliance and the PG was older on average, residual confounding from “healthy adherer” and age effects may inflate PG other-care spend relative to TG.

### Mortality

Mortality was 6.1% in the PG and 5.8% in the TG. Because the PG was older on average, the small difference is likely age-related, and no inference should be made without age-adjusted analysis. Age-stratified results support this interpretation: in every defined age group except “unknown,” mortality was higher in the TG than in the PG. For dogs younger than 10 years (< 1–9 years), the relative risk of death was significantly higher in the TG (*p* < 0.05), consistent with -but not proving—an association with HW disease and/or its treatment. Although the risk was slightly higher in the unknown age group, this finding was not statistically significant. Mortality rates between 0% and 4% in dogs on HW treatment protocols following AHS guidelines have been reported [4], with one study reporting a particularly high mortality rate of 14% [13]. The variation might be owing to inclusion of dogs with different classes of HW diseases, and varying adherences to doxycycline treatment and exercise restriction [4].

In analyses stratified by number of melarsomine injections, mortality was highest among dogs receiving a single injection and declined as the number of injections increased; no deaths were observed in dogs receiving four (*n* = 14) or five (*n* = 5) injections. The elevated mortality in the one-injection group may reflect deaths before a second dose could be given or euthanasia after owners declined further treatment. The absence of deaths in the four- and five-injection groups should be interpreted cautiously owing to the small sample sizes.

### Limitations

Our study has limitations, which are mainly related to the data source, i.e., a transaction database. Clinical outcomes other than mortality were not available and the database did not allow the classification of dogs according to the severity of disease. Therefore, costs and mortality rates could not be split by disease classification, which differentiates between four stages: class 1 (mild), class 2 (moderate), class 3 (severe), and class 4 (caval syndrome) [1]. Because outcomes beyond the treatment period were not evaluated, longer-term health and cost impacts remain uncertain; in surviving dogs, pulmonary hypertension—a frequent, severe sequela—has been reported to persist without improvement at one month after the final adulticide (melarsomine) dose [14].

A principal limitation of the cost analysis is the absence of patient body-weight data, which precluded adjustment for weight-dependent dosing—particularly for melarsomine, a major cost component. This omission introduces size-related confounding: drug costs generally increase with body size, so pooled averages likely overestimate costs for lighter dogs and underestimate costs for heavier dogs. Reported values should therefore be interpreted as unadjusted means across body sizes.

We did not evaluate previous use of heartworm preventive medications in dogs in the TG because the observation period began no earlier than 95 days before the first melarsomine treatment. However, the aim of this study was not to assess the administration or effectiveness of preventive treatment, but rather to evaluate the cost of heartworm disease treatment and prevention. Moreover, the study was not intended to assess treatment success, but to describe treatment patterns in real-life clinical practice. According to American Heartworm Society (AHS) guidance, follow-up after adulticide treatment should include confirmation of a negative heartworm antigen test together with microfilaria testing. Because microfilaria test results were not available in our dataset, we were unable to evaluate treatment clearance according to these criteria.

Despite these limitations, the analysis offers useful estimates to inform clinical and economic decision-making. Future studies should incorporate body weight and longer follow-up to produce size-standardized (or weight-stratified) cost estimates and to address remaining evidence gaps.

## Conclusions

Canine HW disease imposes substantial clinical complexity and cost. In these data, HW disease treatment averaged ~$1457 per case (excluding prevention) versus ~$183 per dog-year for prevention—an ~ eight-fold difference. Coupled with the prolonged, painful treatment course and activity restriction, the evidence supports year-round, lifelong prevention as the medically and economically favored strategy.

## Data Availability

The dataset being basis of our study is subject to copyright by Kynetec. Data supporting the main conclusions of this study are included in the manuscript.

## Footnotes

1 https://www2.census.gov/geo/pdfs/maps-data/maps/reference/us_regdiv.pdf

2 https://www.heartwormsociety.org/veterinary-resources/incidence-maps

3 https://www.avma.org/resources-tools/reports-statistics/us-pet-ownership-statistics

4 https://www.avma.org/news/heartworm-incidence-climbs-despite-preventive-efforts
